# Integrated airway epithelial signaling networks linking allergen-driven inflammation to airway remodeling in asthma

**DOI:** 10.3389/fimmu.2026.1884158

**Published:** 2026-07-20

**Authors:** Shin-Young Park

**Affiliations:** Department of Biotechnology, Pai Chai University, Daejeon, Republic of Korea

**Keywords:** airway epithelium, airway remodeling, allergen-induced signaling networks, corticosteroid resistance, epithelial–mesenchymal transition

## Abstract

Asthma is a chronic inflammatory airway disease characterized by persistent immune activation, airway hyperresponsiveness, and progressive structural remodeling. The airway epithelium functions as the primary sensor of inhaled allergens and actively orchestrates both innate and adaptive immune responses through alarmin and chemokine release, while directly driving structural changes via epithelial–mesenchymal transition and profibrotic mediator production. Upon allergen challenge, exemplified by house dust mite, airway epithelial cells activate an integrated network of signaling pathways, including epidermal growth factor receptor, mitogen-activated protein kinase, PI3K/protein kinase B, nuclear factor kappa B (NF-κB), Janus kinase/signal transducer and activator of transcription (STAT), and transforming growth factor-β/SMAD, which converge on key transcription factors, including NF-κB/RelA, AP-1, STAT6, SMAD2/3, and CCAAT/enhancer-binding protein beta, to coordinately induce the production of acute inflammatory factors, including alarmins (thymic stromal lymphopoietin (TSLP), interleukin (IL)-33, IL-25), and chemokines (C-C motif chemokine ligand 20, C-X-C motif chemokine ligand 8), as well as chronic cellular remodeling, including goblet cell hyperplasia, subepithelial fibrosis, and smooth muscle expansion. This review provides a comprehensive, network-centered synthesis of these mechanisms, with a particular focus on the molecular basis of asthma endotype heterogeneity, amplifying role of viral exacerbations, epigenetic regulation of epithelial inflammatory programs, and signaling basis of corticosteroid resistance. Further, we map the mechanistic basis of the phenotypic heterogeneity across eosinophilic, neutrophilic, and mixed asthma endotypes onto specific network configurations and then discuss therapeutic implications spanning approved biologics, kinase inhibitors, barrier-restoration strategies, and combinatorial approaches.

## 1 Introduction

### 1.1 Asthma as a chronic inflammatory and remodeling airway disease

Asthma affects approximately 300 million people worldwide and is associated with substantial morbidity, disability-adjusted life-years, and healthcare costs (1). It is characterized by episodic airflow obstruction, airway hyperresponsiveness, and chronic airway inflammation (2). For decades, anti-inflammatory pharmacotherapy, principally inhaled corticosteroids, was considered sufficient to control the disease, based on the assumption that structural airway changes were simply downstream consequences of uncontrolled inflammation (3). However, this paradigm has been fundamentally revised. Airway remodeling in asthma includes reticular basement thickening, goblet cell hyperplasia, increased airway smooth muscle mass, and vascular changes, and may develop early in the disease course alongside inflammation, contributing to a persistent airflow limitation not fully reversed by current anti-inflammatory therapy (4–6).

Asthma is immunologically heterogeneous, encompassing Type 2 (T2)-high eosinophilic endotypes driven by interleukin (IL)-4, IL-5, and IL-13 (7, 8), and T2-low neutrophilic or mixed endotypes involving Th1 and Th17 pathways (9, 10). Patients with T2-high asthma often respond well to approved biologics targeting IL-5 (mepolizumab, reslizumab, benralizumab), IL-4Rα (dupilumab), IgE (omalizumab), and TSLP (tezepelumab), whereas T2-low asthma remains a major unmet therapeutic need (11–14). Crucially, the upstream epithelial signals that shape this immune polarization are therefore of direct clinical importance, motivating a network-level analysis of allergen-induced epithelial activation as the organizing framework of this review.

### 1.2 Airway epithelium as an active regulator rather than a passive barrier

The pseudostratified columnar epithelium of the conducting airways is constitutively exposed to inhaled allergens, pollutants, microbial products, and viruses (15, 16). Although the airway epithelium was once regarded as a passive mechanical barrier (17), it is now recognized as the primary immunological interface of the airway (18). This cell layer senses environmental threats through diverse surface and intracellular receptors (19). It integrates these signals through interconnected kinase cascades and orchestrates the downstream immune response via cytokines, chemokines, lipid mediators, and reactive oxygen species (ROS) (11, 20, 21). The immunological state of the airway epithelium, defined by its barrier integrity, receptor expression profile, and epigenetic landscape, is thus a central determinant of whether allergen exposure triggers pathological sensitization or immunological tolerance (19, 22). Critically, the airway epithelium in patients with asthma is intrinsically abnormal: it exhibits reduced tight junction expression, impaired mucociliary differentiation, heightened ROS production, and a pre-activated transcriptional state even in the absence of acute allergen challenge (23, 24). This constitutive dysregulation, shaped by genetic predisposition, environmental exposures, and epigenetic imprinting, lowers the threshold for allergen-induced inflammatory activation and positions the epithelium as both a primary driver and self-sustaining amplifier of asthma pathogenesis (25, 26).

### 1.3 Why integrated epithelial signaling matters: beyond single-pathway models

Mechanistic asthma research has historically focused on individual cytokines, receptors, or signaling pathways in isolation (27, 28). While this approach has provided important insights and enabled the development of effective biologics, it is clear that the biological responses of allergen-exposed airway epithelial cells are not products of discrete linear cascades but of highly interconnected signaling networks with extensive crosstalk, feedback regulation, and emergent properties (29, 30). The same allergen-exposure event simultaneously activates epidermal growth factor receptor (EGFR), multiple toll-like receptors (TLRs), protease-activated receptor-2 (PAR-2), and damage-associated molecular pattern (DAMP) receptor pathways, whose outputs converge on shared kinase modules and transcription factor complexes to induce coordinated cellular responses (16, 20, 29). This integrated signaling network has several important implications. First, it explains the co-induction of inflammatory and remodeling factors by a single cellular stimulus: both acute chemokine production and epithelial-mesenchymal transition (EMT) initiation are driven by overlapping signaling components (31, 32). Second, while targeted biologics have demonstrated substantial clinical efficacy in appropriately selected patients, a subset, particularly those with T2-low, neutrophilic, or treatment-refractory disease, derives limited benefit from any single-pathway approach, consistent with the compensatory capacity of redundant signaling nodes within the epithelial network (32, 33). Third, rational combination strategies targeting multiple network nodes, or single agents targeting true signaling hubs, are cunder preclinical and early-phase clinical investigation as approaches to more comprehensive disease modification, although definitive evidence for superior clinical efficacy over approved monotherapy in asthma has yet to be established (34, 35). Therefore, a network-level understanding of epithelial signaling is not merely of academic interest but is a prerequisite for the next generation of asthma therapeutics.

Numerous reviews have examined individual aspects of airway epithelial biology in asthma, including barrier function, alarmin biology, transforming growth factor-beta (TGF-β)/remodeling, or specific signaling pathways (29, 36–38). In contrast, the present review conceptualizes epithelial signaling as an interconnected network rather than a collection of discrete pathways. By consolidating current evidence, it delineates how diverse signaling inputs converge within the allergen-exposed airway epithelium to drive the coordinated induction of acute inflammatory responses and chronic airway remodeling. Beyond this network-level perspective, the review also addresses several important dimensions relatively underrepresented in prior studies, including: i) epithelial signaling mechanisms underlying asthma endotype heterogeneity; ii) biology of viral exacerbations and their interaction with allergen-driven pathways; iii) epigenetic regulation of epithelial inflammatory programs; and iv) molecular basis of corticosteroid resistance. Finally, it discusses the therapeutic implications and key priorities for future research. Existing reviews have addressed individual aspects of epithelial biology in depth, including barrier dysfunction, alarmin release, TGF-β–driven remodeling, and specific kinase pathways, but have not framed these components as part of a unified epithelial network in which the same signaling architecture simultaneously generates both inflammatory and structural outputs. The present review addresses this gap by: (i) systematically mapping the crosstalk and mutual reinforcement among EGFR, MAPK, PI3K/Akt, NF-κB, JAK/STAT, and TGF-β/SMAD within a single integrated framework; (ii) positioning C/EBPβ as a transcriptional integrator of converging pathway outputs that selectively regulates chemokine programs not fully explained by NF-κB or AP-1 alone; (iii) analyzing asthma endotypes as alternative configurations of a shared epithelial network rather than immunologically distinct diseases; and (iv) deriving therapeutic implications from the topology of the network—including redundancy, crosstalk, and hub architecture—rather than from single-target logic. These four elements collectively constitute the primary conceptual contribution of this review. Accordingly, this review focuses primarily on allergen-driven, T2-high asthma as the best-characterized model of epithelial signaling network activation; non-allergic and T2-low endotypes are discussed where mechanistic contrasts illuminate endotype-specific network configurations, but a comprehensive treatment of non-allergic asthma pathogenesis is beyond the scope of the present work.

## 2 Airway epithelium as the first responder to inhaled allergens

### 2.1 Cellular composition and structural roles of airway epithelial cells

The airway epithelium is a pseudostratified columnar epithelium composed of several functionally distinct cell populations (39, 40). Ciliated columnar cells constitute the majority of the surface epithelium and play a central role in mucociliary clearance (39). Goblet cells produce the gel-forming mucins MUC5AC and MUC5B (41). Club cells secrete club cell secretory protein 16 and contribute to the maintenance of epithelial homeostasis (26, 42). Basal cells serve as multipotent progenitors capable of differentiating into the other major epithelial cell types (43). Single-cell RNA sequencing studies have further expanded the known cellular diversity of the airway epithelium by identifying additional specialized populations (44). Among these, tuft cells, also known as brush cells, are chemosensory epithelial cells characterized by their expression of taste receptors (TAS1R, TAS2R) (45). Ionocytes, which highly express cystic fibrosis transmembrane conductance regulator and Forkhead box I1, are involved in the regulation of airway surface liquid composition (46, 47). Most recently, a ‘hillock’ cell population has been described and is thought to represent an intermediate differentiation state with distinct secretory features (48).

### 2.2 Major inhaled allergens and shared mechanisms of epithelial activation

The major inhaled allergens include house dust mite (HDM), cockroach, fungal allergens, such as *Alternaria alternata*; grass pollens, and animal dander derived from cats and dogs (49, 50). Among these, HDM is the most extensively studied and clinically important inhalant allergen worldwide, although cockroach, molds, pollens, and pet-derived allergens also contribute substantially to allergic airway disease (51, 52). Despite marked differences in biochemical composition, these aeroallergens share several common mechanisms of epithelial activation. These include protease-mediated disruption of tight junctions and activation of PAR-2, engagement of pattern recognition receptors (PRRs) by structurally associated microbial or polysaccharide components, and induction or amplification of ROS generation in airway epithelial cells (53, 54). Collectively, these processes lower the threshold for epithelial alarmin signaling and promote downstream inflammatory responses (16, 55). Among inhaled allergens, HDM model provides the best characterized model for investigating these integrated epithelial responses and is therefore discussed in detail in the following section (51, 56).

While these convergent mechanisms provide a useful conceptual framework, allergen-specific differences in protease class, activity level, biochemical composition, co-delivered environmental factors, and exposure characteristics result in quantitative and qualitative differences in epithelial activation profiles that should not be overlooked. Der p 1 is a highly potent cysteine protease whose barrier-disruptive activity substantially exceeds that of cockroach allergens, which activate epithelial cells predominantly through serine protease-dependent PAR-2 engagement (57). Fungal allergens such as *Alternaria alternata* trigger IL-33 secretion from airway epithelial cells primarily through ROS-mediated mechanisms (58), with additional contributions from serine protease activity via PAR-2 and ATP signaling (59). Co-delivered environmental contaminants, including LPS levels associated with high-endotoxin environments or particulate matter, further modulate TLR4-dependent signal amplification in an exposure context-dependent manner. Seasonality and cumulative sensitization history add further complexity by altering the threshold for epithelial alarmin release. Collectively, these allergen-specific nuances underscore that while the framework described here is broadly applicable, its quantitative parameters and relative pathway contributions require calibration for each sensitization context.

### 2.3 HDM as the prototypic epithelial-activating allergen

**Figure 1:**
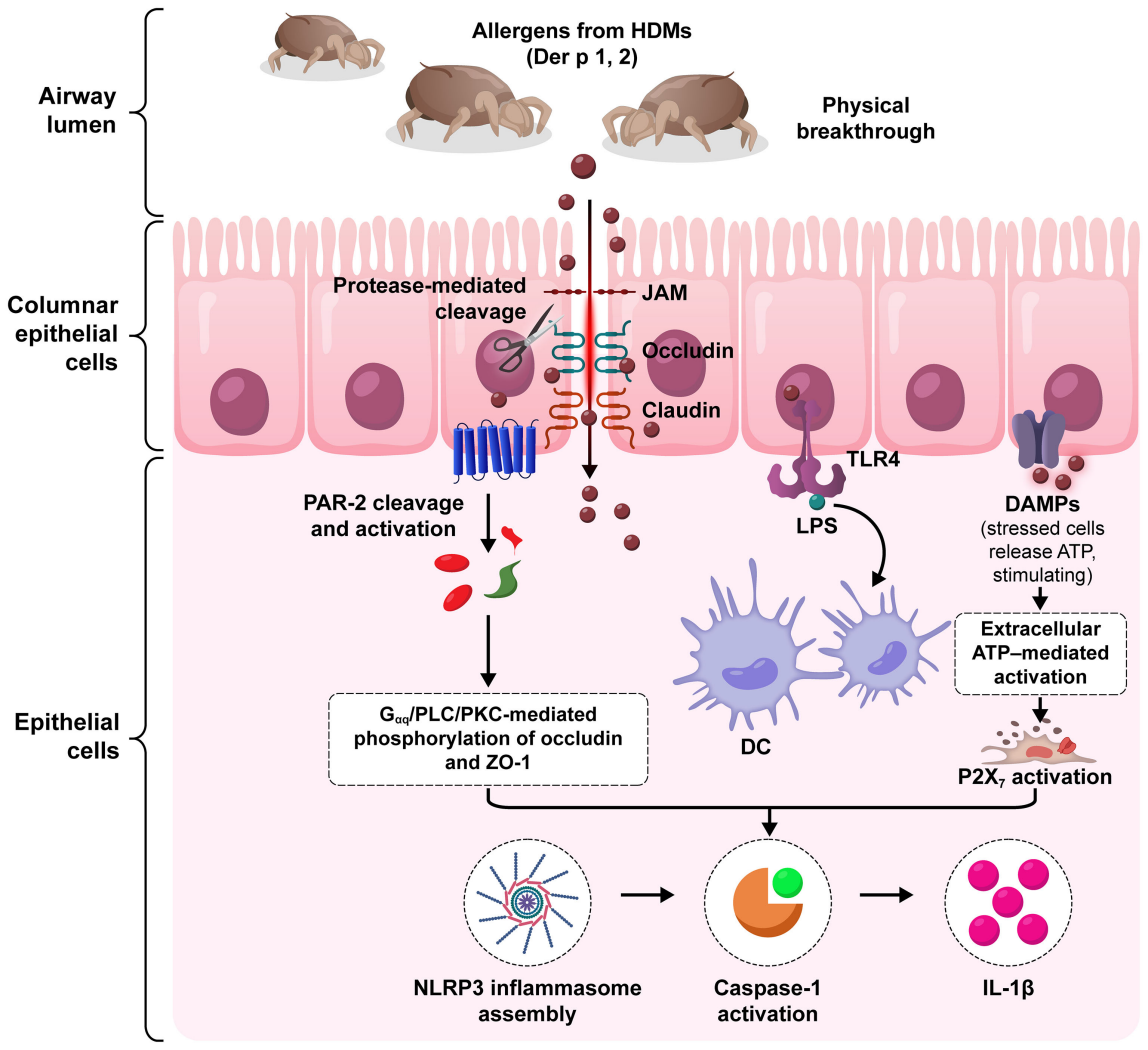
House dust mite–induced epithelial barrier disruption and inflammasome activation in the asthmatic airway. House dust mite (HDM) allergens, including Der p 1 and Der p 2, disrupt airway epithelial barrier integrity through both physical and protease-mediated mechanisms. Proteolytic cleavage of junctional proteins, including junctional adhesion molecule (JAM), occludin, and claudin, facilitates epithelial breach, while protease-activated receptor-2 (PAR-2) activation promotes Gαq/phospholipase C/protein kinase C-dependent phosphorylation of occludin and zonula occludens-1 (ZO-1), further destabilizing intercellular junctions. In parallel, lipopolysaccharide (LPS)-associated Toll-like receptor 4 (TLR4) signaling and damage-associated molecular patterns (DAMPs) released from stressed epithelial cells stimulate extracellular adenosine triphosphate (ATP)-mediated P2X7 activation, leading to NLR family pyrin domain containing 3 (NLRP3) inflammasome assembly, caspase-1 activation, and interleukin-1β (IL-1β) production. Collectively, this figure illustrates how HDM exposure initiates epithelial barrier failure and early innate inflammatory signaling.

The two clinically dominant HDM species, *Dermatophagoides pteronyssinus* (Der p) and *D. farinae* (Der f), possess overlapping but distinct allergen repertoires that collectively engage multiple epithelial sensing pathways (60). Der p 1, the major cysteine protease of *D. pteronyssinus*, disrupts epithelial barrier integrity by cleaving tight junction proteins, including occludin and claudin-1, thereby increasing paracellular permeability (61). Der p 1 also activates PAR-2 and exerts additional immunomodulatory effects by cleaving CD23 on B cells and CD25 on regulatory T cells (62, 63). Its functional homologue, Der f 1, acts through a similar cysteine protease-dependent mechanism and exerts comparable barrier-disruptive effects in human bronchial epithelial models, although differences in enzymatic kinetics may contribute to species-specific biological activity (64, 65). In addition, Der p 2 and Der f 2 enhance TLR4 signaling through myeloid differentiation factor 2 (MD-2) mimicry in the presence of environmental lipopolysaccharide (LPS) (66, 67). Chitin-containing components further contribute to epithelial activation through TLR2-associated pathways and induction of IL-33 release (68, 69). Additional *D. farinae* allergens, including Der f 3 and Der f 6, reinforce protease-dependent PAR-2 activation and barrier dysfunction (70, 71). Together, these features establish HDM as a prototypic allergen for mechanistic studies of epithelial activation in allergic airway disease. These integrated mechanisms of HDM-induced epithelial activation are summarized in **Figure 1**.

### 2.4 Early epithelial responses to allergen exposure

**Figure 2:**
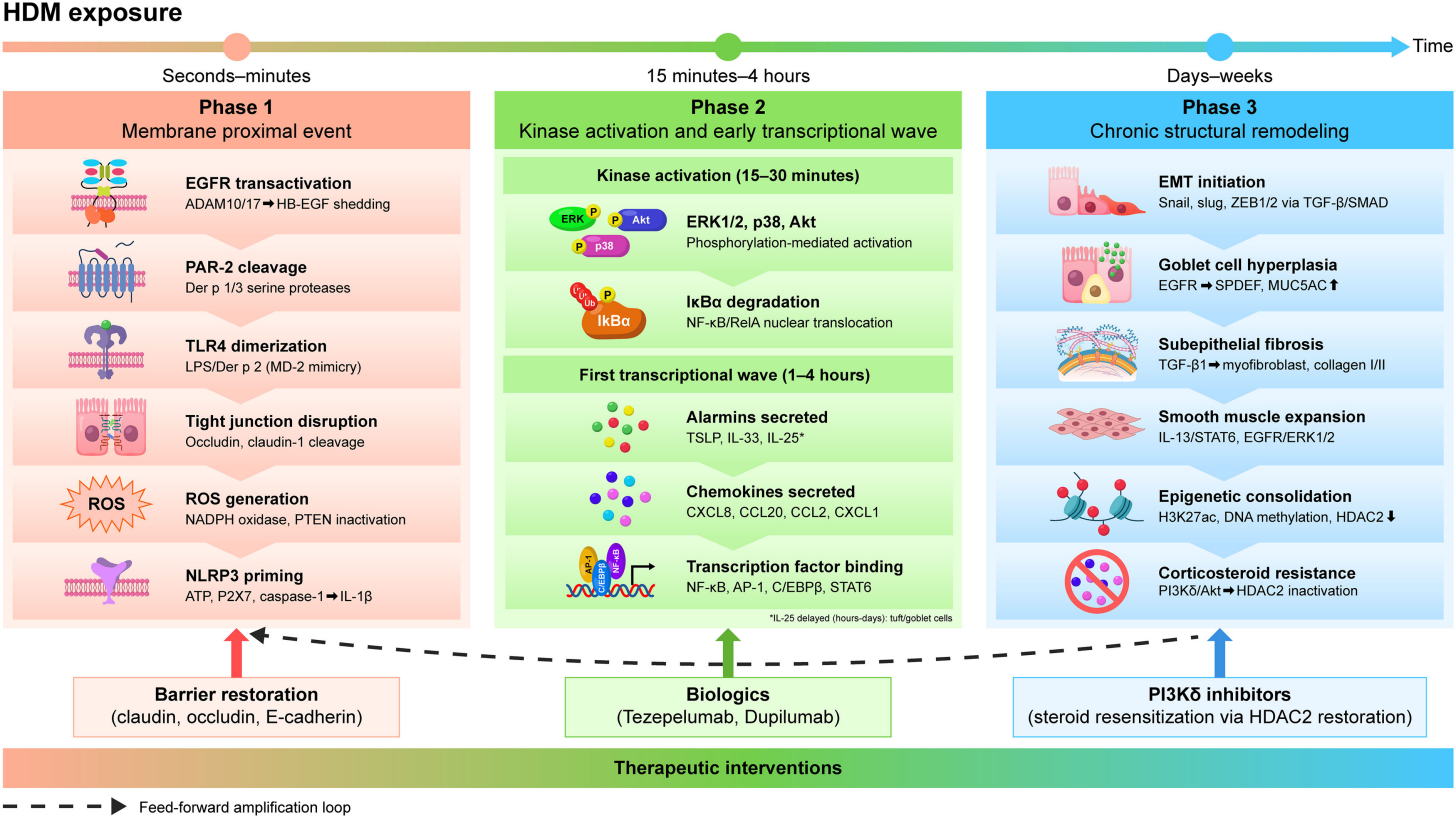
Temporal progression of allergen-induced airway epithelial responses from initial HDM exposure to chronic structural remodeling. House dust mite (HDM) allergen exposure triggers a temporally structured sequence of airway epithelial responses organized across three mechanistically distinct phases. Phase 1 (seconds to minutes) encompasses membrane-proximal events: EGFR transactivation via ADAM10/17-mediated HB-EGF shedding, PAR-2 cleavage by Der p 1/3 serine proteases, TLR4 dimerization facilitated by LPS and Der p 2 MD-2 mimicry, proteolytic disruption of tight junction proteins (occludin and claudin-1), NADPH oxidase-mediated ROS generation with oxidative PTEN inactivation, and NLRP3 inflammasome priming via ATP/P2X7 signaling leading to caspase-1 activation and IL-1β maturation. Phase 2 (15 minutes to 4 hours) comprises intracellular kinase activation, including ERK1/2, p38, and Akt phosphorylation-and IκBα proteasomal degradation enabling NF-κB/RelA nuclear translocation, followed by the first transcriptional wave: secretion of alarmins (TSLP, IL-33, and IL-25[the latter delayed hours to days due to tuft/goblet cell origin]), and chemokines (CXCL8, CCL20, CCL2, CXCL1), with cooperative occupancy of inflammatory gene promoters by NF-κB, AP-1, C/EBPβ, and STAT6. Phase 3 (days to weeks) represents chronic structural remodeling: EMT initiation via TGF-β/SMAD-driven Snail/Slug/ZEB1/2 induction; EGFR/SPDEF-mediated goblet cell hyperplasia with MUC5AC upregulation; TGF-β1-driven subepithelial fibroblast to myofibroblast differentiation and collagen I/III deposition; IL-13/STAT6- and EGFR/ERK1/2-mediated airway smooth muscle expansion; epigenetic consolidation through H3K27 acetylation and DNA methylation; and PI3Kδ/Akt-mediated HDAC2 inactivation underlying corticosteroid resistance. A feed-forward amplification loop (dashed arrow) indicates that Phase 3 structural changes perpetuate Phase 1 and 2 signaling by sustaining barrier dysfunction and epigenetically consolidated inflammatory programs. Key therapeutic intervention points are indicated at the bottom: barrier restoration strategies targeting Phase 1 junction proteins (claudin, occludin, E-cadherin); approved biologics tezepelumab and dupilumab targeting Phase 2 alarmin and cytokine output; and PI3Kδ inhibitors targeting Phase 3 corticosteroid resistance via HDAC2 restoration. Abbreviations: ADAM, a disintegrin and metalloproteinase; C/EBPβ, CCAAT/enhancer-binding protein beta; EMT, epithelial–mesenchymal transition; HB-EGF, heparin-binding EGF-like growth factor; IκBα, inhibitor of kappa B alpha; MUC5AC, mucin 5AC; NLRP3, NLR family pyrin domain containing 3; SPDEF, SAM pointed domain-containing ETS transcription factor; ZEB, zinc finger E-box-binding homeobox.

The temporal sequence of airway epithelial responses to allergen exposure spans several orders of magnitude (16). Within seconds to minutes, membrane-proximal events occur: EGFR transactivation through a disintegrin and metalloproteinase domain-containing protein 10/17 (ADAM10/17)-mediated heparin-binding EGF-like growth factor (HB-EGF) shedding, PAR-2 cleavage, TLR4 dimerization, myeloid differentiation primary response 88 recruitment, and tight junction disruption by Der p 1 (20, 72). Moreover, within 15–30 minutes, extracellular signal-regulated kinase 1/2 (ERK1/2), p38 mitogen-activated protein kinase (p38 MAPK), and protein kinase B (Akt) are phosphorylated, and inhibitor of kappa B alpha (IκBα) undergoes proteasomal degradation, allowing nuclear factor kappa B (NF-κB)/RelA dimers to occupy target gene promoters (73, 74). Further, within 1–4 hours, the first wave of transcriptional output is produced: thymic stromal lymphopoietin (TSLP), IL-33, C-X-C motif chemokine ligand (CXCL) 8/IL-8, C-C motif chemokine ligand (CCL) 20, CCL2, and CXCL1 are secreted. Additionally, IL-25 production from tuft cells is delayed (hours to days) relative to that of TSLP and IL-33 (75, 76). This temporal hierarchy has immunological significance: IL-33 and TSLP act rapidly on resident ILC2s and mast cells to induce an immediate innate type 2 response, while IL-25 preferentially drives later amplification of adaptive Th2 responses (75). Phase 3 chronic structural outputs, including EMT initiation, goblet cell hyperplasia, subepithelial fibrosis, smooth muscle expansion, epigenetic consolidation, and corticosteroid resistance, are driven by the sustained signaling networks described in Sections 4 through 7. The full temporal hierarchy of these responses, spanning membrane-proximal events, kinase activation and first transcriptional wave, and chronic structural remodeling outputs, is illustrated schematically in **Figure 2**.

## 3 Allergen sensing and epithelial barrier dysfunction

### 3.1 Epithelial barrier integrity in healthy vs. asthmatic airways

Tight junction integrity in healthy airways is maintained by the claudin family, occludin, tricellulin, and junction adhesion molecules (A/B/C), scaffolded by zonula occludens proteins (ZO-1/2/3) (77). Adherens junctions are maintained by E-cadherin–β-catenin–α-catenin complexes (78). Together, these induce a transepithelial electrical resistance (TEER) of 200–800 Ω·cm² in differentiated bronchial epithelial cultures (79).

In asthmatic airways, reduced and discontinuous expression of claudin-1, claudin-18, occludin, and E-cadherin at epithelial cell interfaces is consistently observed in bronchial biopsies, with TEER measurements in human bronchial epithelial cells (HBECs) from asthmatic donors showing 20–40% lower resistance than those in healthy controls even in the absence of allergen challenge (80, 81). Genetic association studies have identified variants in filaggrin, desmoglein-1, and serine protease inhibitors as asthma risk factors that directly impair barrier function, establishing barrier dysfunction as a primary predisposing factor rather than solely a secondary consequence of inflammation (82, 83).

### 3.2 Tight junction disruption and enhanced permeability

Der p 1-induced tight junction disruption begins with the cleavage of the extracellular domains of occludin and claudin-1, followed by redistribution of ZO-1 from tight junction plaques to the cytoplasm and a measurable TEER decrease within 30–60 minutes (82, 84). Simultaneously, PAR-2 activation initiates Gαq/phospholipase C (PLC)/protein kinase C (PKC)-mediated phosphorylation of occludin and ZO-1, further disassembling tight junction complexes (85). Type 2 cytokines perpetuate this dysfunction: IL-4 and IL-13, acting through Janus kinase 1 (JAK1)/signal transducer and activator of transcription 6 (STAT6), downregulate claudin-1, claudin-18, and occludin while upregulating the leaky claudin-2 (86, 87). Tumor necrosis factor-alpha (TNF-α) activates myosin light chain kinase, and IL-17A promotes tight junction disruption through RhoA/ROCK-mediated cytoskeletal contraction (88, 89). The resulting leaky epithelium facilitates allergen penetration to subepithelial antigen-presenting cells, amplifying sensitization in a feed-forward cycle (23).

### 3.3 Protease-dependent and protease-independent allergen sensing

PAR-2 activation by HDM serine proteases (Der p 3 and Der p 9) initiates Gαq/PLC/inositol 1,4,5-triphosphate/diacylglycerol signaling, leading to PKC activation and subsequently MAPK and NF-κB activation (90, 91). PAR-2 also signals through β-arrestin-mediated EGFR transactivation, providing a non-G-protein-dependent route to MAPK activation (92). PRR-mediated sensing involves TLR4 (activated by HDM-associated LPS and Der p 2-facilitated MD-2 mimicry), TLR2 (recognizing HDM chitin-associated lipopeptides), dectin-1 (recognizing β-glucan from mold allergens), and NOD-like receptor family pyrin domain containing 3 (NLRP3, activated by uric acid, ATP, and particulate allergen components) (93–95). Activated NLRP3 assembles the canonical inflammasome, activating caspase-1 to process pro-IL-1β and pro-IL-18, and gasdermin D to form plasma membrane pores facilitating non-classical cytokine secretion (96, 97).

### 3.4 Pattern recognition receptor- and danger-associated signaling

Beyond PRR activation by exogenous allergen-associated patterns, airway epithelial cells release endogenous DAMPs upon barrier disruption that amplify the danger signal (17, 22). Extracellular ATP activates P2X7 receptors to provide the second signal for NLRP3 inflammasome assembly (98). High mobility group box 1, released by stressed epithelial cells, activates TLR4 and receptor for advanced glycation end products on neighboring cells, amplifying NF-κB activation (99). IL-1α, also constitutively stored in epithelial nuclei (analogously to IL-33), is released upon necrotic cell death to perpetuate NF-κB-driven inflammatory signaling through the IL-1 receptor type 1/IL-1 receptor accessory protein receptor complex (100, 101).

### 3.5 Barrier dysfunction as a trigger for downstream inflammatory cascades

Tight junction and adherens junction disruption is itself a potent trigger of intracellular signaling (102). E-cadherin disruption releases β-catenin, allowing its nuclear translocation and activation of T cell factor/lymphoid enhancer-binding factor target genes associated with EMT (103). Disruption of tight-junction scaffolding, including claudin/ZO-1-associated complexes, engages FAK/Src-dependent signaling and can propagate PI3K/Akt and MAPK activation independently of canonical growth factor receptor inputs (104–106). ROS generated through NADPH oxidase activation and mitochondria activate NF-κB and MAPK cascades while oxidatively inactivating phosphatase and tensin homolog, thereby favoring sustained PI3K/Akt-dependent pro-inflammatory signaling (107, 108).

## 4 Core intracellular signaling pathways in airway epithelial cells

### 4.1 EGFR signaling in epithelial activation and repair

Epidermal growth factor receptor (EGFR; ErbB1/HER1) functions as a central signaling hub in the asthmatic airway epithelium, integrating extracellular injury signals with intracellular programs that regulate epithelial activation, repair, and pathological remodeling (109). EGFR is activated by epidermal growth factor (EGF) family ligands, including EGF, TGF-alpha (TGF-α), amphiregulin (AREG), epiregulin, HB-EGF, and epigen, shed from the cell surface by ADAM10 and ADAM17 in response to allergen exposure and PAR-2 activation (110–112). Upon activation, EGFR undergoes autophosphorylation at Y1068 and Y1086, generating src-homology 2 docking sites that recruit growth factor receptor-bound protein 2/son of sevenless that activate the RAS/RAF/MEK/ERK pathway, PLCγ that triggers PKC activation and calcium release, and p85 regulatory subunit of PI3K that initiates PI3K/Akt signaling (113).

In asthmatic airways, EGFR expression is elevated and correlates with goblet cell density and reticular basement membrane thickness, consistent with its roles in driving goblet cell metaplasia through ERK1/2-mediated SAM pointed domain-containing ETS transcription factor induction and fibrosis through TGF-β1 paracrine signaling (114, 115). A feed-forward mechanism sustains EGFR activation: NF-κB transcriptionally induces TGF-α and HB-EGF, while ERK1/2 induces AP-1-dependent AREG expression, creating autocrine EGFR activation loops (116). ErbB2 (HER2), upregulated in asthmatic epithelium, amplifies EGFR signaling through allosteric mechanisms (117). Accordingly, EGFR should be viewed not simply as a repair receptor, but as a central amplifier that links epithelial injury sensing to persistent inflammatory and remodeling responses in asthma.

The remodeling consequences of sustained EGFR activation are therefore mechanistically direct: ERK1/2-mediated SPDEF induction drives goblet cell trans differentiation and mucous metaplasia (114, 115), while EGFR-stimulated TGF-β1 paracrine release activates subepithelial fibroblasts to initiate collagen deposition and reticular basement membrane thickening (109, 118). This positions EGFR not merely as a repair receptor but as a direct mechanistic bridge between acute epithelial injury sensing and the structural remodeling hallmarks of chronic asthma.

From a therapeutic standpoint, EGFR tyrosine kinase inhibitors, including gefitinib and afatinib, have demonstrated attenuation of goblet cell metaplasia, mucin overproduction, and airway hyperresponsiveness in allergen-challenged preclinical asthma models; however, clinical translation remains constrained by the systemic toxicity profile established in oncology indications, and no dedicated asthma trials have been completed to date (109, 115, 119).

### 4.2 MAPK pathways: ERK1/2, c-Jun N-terminal kinase, and p38

Mitogen-activated protein kinase (MAPK) pathways constitute a major downstream arm of epithelial signaling in asthma and mediate the conversion of receptor activation into inflammatory gene expression, stress adaptation, and epithelial plasticity (120). Among these pathways, ERK1/2 are activated downstream of EGFR/RAS and by multiple upstream inputs, including PAR-2, TLR4, and inflammatory cytokines through the canonical RAF/MEK1/2/ERK1/2 cascade (121). ERK1/2 phosphorylate the AP-1 components c-Fos (through ternary complex factor family member Elk-1) and ribosomal S6 kinase 1/2, and cooperate with NF-κB at composite NF-κB/AP-1 regulatory elements to drive synergistic induction of inflammatory genes, including *CXCL8*, *CCL2*, *CCL5*, *CCL20*, granulocyte-macrophage colony-stimulating factor (*GM-CSF*), and *MUC5AC (*121–123). JNK is activated by cellular stress, including ROS, through MKK4/7 downstream of ASK1 and MLK3, phosphorylating c-Jun and contributing to epithelial cell shedding (124). p38 MAPK, predominantly the α and β isoforms, is activated through the TRAF2/6–TAK1–MKK3/6 cascade downstream of IL-1R/TLR signaling, and through MAPK-activated protein kinases 2/3 (MK2/3), it enhances the stability of AU-rich element-containing mRNAs encoding TNF-α, IL-6, CXCL8, and COX-2, broadly amplifying post-transcriptional cytokine expression (125, 126). Among MAPK isoforms, asthma-relevant functional evidence is strongest for p38α (MAPK14) and ERK1/2 in airway epithelial cells; p38α is the predominant isoform in bronchial epithelium and the principal target of clinical p38 inhibitor programs in airway inflammatory disease (127, 128). In contrast to the isoform-selective dominance of PI3Kδ, MAPK signaling contributes to asthmatic epithelial activation across multiple isoforms, with the predominant isoform and relative contribution varying by cellular context, stimulus type, and disease severity. From a therapeutic standpoint, the p38α inhibitor losmapimod has been evaluated in Phase II trials in chronic obstructive pulmonary disease, with overall negative results for the primary clinical endpoints; *post hoc* subgroup analyses have raised the hypothesis of restored corticosteroid responsiveness in low-eosinophil populations, although this signal has not yet been prospectively validated in asthma (127, 129, 130).

### 4.3 PI3K/Akt signaling in survival, inflammation, and remodeling

The PI3K/Akt pathway provides a complementary signaling axis through which the asthmatic epithelium integrates survival, inflammatory, and remodeling cues (131). Class I PI3Kα (p110α/p85) and PI3Kδ (p110δ/p85) are the principal isoforms activated in allergen-exposed epithelial cells, generating phosphatidylinositol (3–5)-trisphosphate, which recruits Akt to the membrane for 3-phosphoinositide-dependent protein kinase-1-mediated Thr308 and mTORC2-mediated Ser473 phosphorylation (131–133). Akt activates IKKα, leading to NF-κB activation, promotes mTORC1-dependent translation of cytokine mRNAs through TSC2 phosphorylation, and stabilizes Snail and Myc through GSK-3β inhibition, driving EMT and proliferation (131, 134). PTEN is transcriptionally suppressed by NF-κB, epigenetically silenced, and post-translationally inactivated by ROS in asthmatic epithelium, collectively amplifying PI3K/Akt pathway activity (134). The PI3Kδ isoform is of particular therapeutic interest, being enriched in hematopoietic and epithelial cells with anti-asthmatic effects in preclinical models (132). PI3K/Akt–mediated Snail stabilization and GSK-3β inhibition represent the mechanistic link between allergen-triggered intracellular signaling and the initiation of EMT (131, 134), with downstream consequences that include loss of E-cadherin, acquisition of vimentin and fibronectin, and the deposition of subepithelial extracellular matrix components that characterize structural airway remodeling in asthma (135, 136).

**Figure 3:**
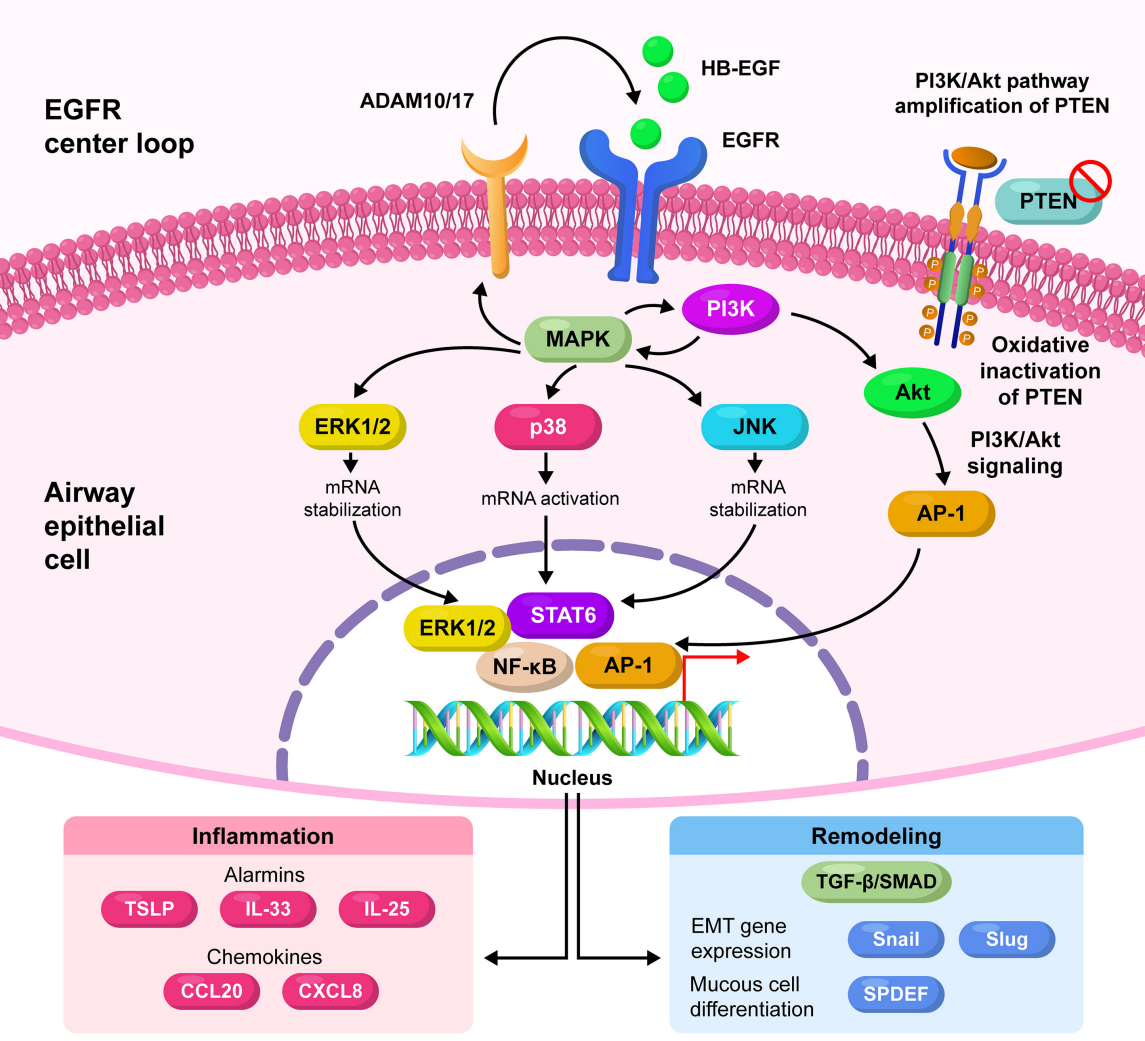
Epidermal growth factor receptor-centered epithelial signaling network linking inflammation to airway remodeling. Airway epithelial injury activates an epidermal growth factor receptor (EGFR)-centered signaling loop in which a disintegrin and metalloproteinase 10/17 (ADAM10/17)-mediated shedding of heparin-binding epidermal growth factor-like growth factor (HB-EGF) promotes EGFR activation. Downstream mitogen-activated protein kinase (MAPK) and phosphoinositide 3-kinase/protein kinase B (PI3K/Akt) signaling amplify nuclear transcriptional responses through nuclear factor-κB (NF-κB), activator protein-1 (AP-1), and signal transducer and activator of transcription 6 (STAT6). Oxidative inactivation of phosphatase and tensin homolog (PTEN) further enhances PI3K/Akt signaling. These integrated pathways induce epithelial production of alarmins and chemokines, including thymic stromal lymphopoietin (TSLP), interleukin-33 (IL-33), interleukin-25 (IL-25), CCL20, and CXCL8, while concurrently promoting remodeling-associated programs such as transforming growth factor-β/SMAD (TGF-β/SMAD) signaling, epithelial–mesenchymal transition (EMT), and mucous cell differentiation.

Within the asthmatic airway, PI3K/Akt signaling therefore occupies a strategic position at the intersection of inflammatory amplification, epithelial survival, and remodeling-associated plasticity. As summarized in **Figure 3**, this EGFR-centered epithelial signaling network provides a mechanistic framework linking upstream epithelial injury sensing to downstream inflammatory amplification and airway remodeling. The PI3Kδ-selective inhibitor nemiralisib has been evaluated in a randomized controlled trial in persistent uncontrolled asthma, although further development for this indication was subsequently discontinued owing to limited efficacy; idelalisib, an oncology-approved PI3Kδ inhibitor, has demonstrated anti-inflammatory effects in preclinical airway models, supporting PI3Kδ as a mechanistically tractable target whose clinical validation in asthma nonetheless remains incomplete (131, 132, 137).

### 4.4 NF-κB signaling in inflammatory gene expression

NF-κB signaling represents a major transcriptional endpoint through which diverse epithelial signaling pathways converge to drive inflammatory gene expression in asthma (138). The canonical NF-κB activation pathway involves IκB kinase (IKK) complex activation downstream of TNF receptor-associated factor 6 (TLR/IL-1R signaling), receptor-interacting protein kinase 1 (TNF-R signaling), and CARMA3 (GPCR/PAR-2 signaling) (139, 140). IKKβ-mediated IκBα phosphorylation at Ser32/36 targets it for proteasomal degradation, releasing p65/p50 dimers for nuclear translocation (140). NF-κB target genes in allergen-activated epithelial cells include those encoding cytokines (*TNF*-α, *IL-1β*, *IL*-6, *GM-CSF*), chemokines (*CXCL1/2/3/5/8*, *CCL2/5/17/20/22*), adhesion molecules (*ICAM-1*, *VCAM-1*), enzymes (*COX*-2, *iNOS*, *MMP*-1/3/9), growth factors (*TGF-α, HB-EGF, VEGF*), and anti-apoptotic proteins (*BCL*-2, *BCL*-*xL*) *(*74, 141, 142). In chronic asthma, the normal NF-κB negative feedback through IκBα is impaired by persistent upstream activation, epigenetic modification of the IκBα promoter, and iNOS-derived NO-mediated IκBα nitrosylation, sustaining constitutive low-grade NF-κB activity (138, 143). Pharmacological IKK-2 inhibitors (e.g., IMD-0354, TPCA-1, PHA-408) and NF-κB decoy oligonucleotide strategies have shown proof-of-concept efficacy in preclinical asthma models; clinical translation, however, remains limited by the systemic toxicity concerns associated with broad NF-κB suppression, given the homeostatic role of this pathway in host defense and tissue integrity (138–140, 144, 145).

### 4.5 JAK/STAT signaling: amplification and endotype determination

Janus kinase/signal transducer and activator of transcription (JAK/STAT) signaling provides an additional layer through which epithelial-derived and immune-derived cytokines shape disease phenotype in asthma (146). Among the STAT family members, STAT6, activated by IL-4/IL-13 via JAK1/TYK2 and JAK1/JAK2, respectively, drives the type 2 transcriptional program, including MUC5AC/goblet cell differentiation, CCL11/CCL26 eosinophil chemo-attractants, CCL17/CCL22 Th2 chemo-attractants, periostin, and CLCA1, while repressing type 1 inflammatory genes (147, 148). STAT3, activated by IL-6, IL-10, and IL-33, promotes anti-apoptotic gene expression and goblet cell terminal differentiation (147, 149). STAT1, activated downstream of IFN-γ and type I/III interferons, upregulates antiviral and anti-proliferative programs (150). In T2-high asthma, dominant STAT6 activity suppresses STAT1 through STAT6-induced suppressor of cytokine signaling 1, while neutrophilic/severe asthma features stronger STAT1 and STAT3 activity—a molecular determinant of endotype and therapeutic response (150, 151). Within the JAK/STAT axis, inhaled JAK1-selective inhibitors (AZD0449, AZD4604) have entered early-phase clinical development for asthma, with formulations designed to maximize airway efficacy while minimizing systemic exposure (152, 153); oral JAK inhibitors such as ruxolitinib and abrocitinib, currently approved for myelofibrosis and atopic dermatitis (146), respectively, have demonstrated preclinical efficacy in allergic airway and food allergy models but have not advanced into dedicated asthma trials, owing to concerns regarding systemic immunosuppression.

### 4.6 TGF-β/SMAD signaling in profibrotic remodeling

TGF-β/SMAD signaling is a central driver of the profibrotic and remodeling phenotype of the asthmatic airway epithelium (154). Active TGF-β1 binds to the heterotetrameric TGF-β receptor complex (TGF-βRII/ALK5), inducing SMAD2/3 phosphorylation (155). Phospho-SMAD2/3 associates with SMAD4 and translocates to the nucleus to induce EMT-related transcription factors (Snail, Slug, Twist1, ZEB1/2), ECM proteins (collagen I/III, fibronectin, tenascin-C), and anti-fibrinolytic proteins (PAI-1, TIMP-1) (135, 136, 156, 157). Inhibitory SMADs (SMAD6/7) provide negative feedback through SMURF1/2-mediated ALK5 ubiquitination (158). Non-SMAD TGF-β signaling through TAK1-mediated p38 MAPK and NF-κB, ShcA-dependent ERK1/2, and PI3K/Akt is particularly important for the rapid cytoskeletal reorganization preceding transcription-dependent EMT (159). TGF-β1 also acts on subepithelial fibroblasts and smooth muscle cells to drive myofibroblast differentiation and ECM production, making it the central soluble mediator linking epithelial injury to mesenchymal remodeling (118).

The structural remodeling outputs of TGF-β/SMAD signaling are therefore both epithelial and mesenchymal in nature: within the epithelium, SMAD2/3-driven transcription of Snail, Slug, and ZEB1/2 initiates EMT with loss of E-cadherin and acquisition of vimentin and fibronectin (135, 157); in the subepithelial compartment, TGF-β1 secreted by allergen-activated epithelial cells activates resident fibroblasts, driving their differentiation into α-SMA-positive myofibroblasts that deposit collagen I/III and tenascin-C to form the thickened reticular basement membrane (118, 136, 156); and in airway smooth muscle, TGF-β1 acts synergistically with IL-13 to promote hypertrophy and proliferative expansion (160). This epithelial-to-mesenchymal paracrine axis positions TGF-β/SMAD signaling as the central mechanistic bridge linking allergen-triggered epithelial network activation to the irreversible structural changes that define severe, progressive asthma (154). Anti-TGF-β strategies have demonstrated anti-fibrotic effects in preclinical airway remodeling models, including the pan-TGF-β neutralizing monoclonal antibody fresolimumab (evaluated in Phase I/II trials in idiopathic pulmonary fibrosis and systemic sclerosis), the IPF-approved small molecule pirfenidone, and ALK5 (TGF-β receptor type I) kinase inhibitor such as SB-505124 employed as mechanistic tools; dedicated clinical development targeting asthma-specific airway remodeling, however, remains an unmet need (37, 38, 161).

### 4.7 CCAAT/enhancer-binding protein beta and transcriptional integration of epithelial inflammation

C/EBPβ is a bZIP transcription factor giving rise to three isoforms through differential translation initiation: the transcriptional activators LAP1 and LAP2, and the dominant-negative inhibitor LIP (162). C/EBPβ is rapidly induced following allergen exposure through ERK1/2-mediated phosphorylation at Thr188 and STAT3-dependent mRNA induction downstream of IL-6 (163, 164). C/EBPβ induces CCL20 expression in an ERK1/2-dependent manner, with C/EBPβ knockdown more effectively blocking CCL20 induction than NF-κB inhibition (93). ChIP-seq studies demonstrate that C/EBPβ cooperatively binds to promoters and enhancers with NF-κB, AP-1, and STAT3, regulating selective subsets of inflammatory target genes (165, 166). C/EBPβ currently lacks a selective clinical-stage inhibitor; nevertheless, its position as a downstream integrator of ERK1/2 and STAT3 signaling suggests that indirect targeting through upstream kinase inhibition may attenuate C/EBPβ-dependent chemokine programs, including the HDM-induced CCL20 axis recently implicated in epithelial-mesenchymal transition and chronic airway inflammation (93, 163, 164).

### 4.8 Pathway integration and crosstalk: the basis of network behavior

The signaling pathways described in the preceding sections do not operate as independent linear cascades but function as a densely interconnected network with extensive crosstalk at multiple levels. EGFR serves as a convergence point for PAR-2–, TLR4–, and DAMP-initiated signals, feeding simultaneously into both MAPK and PI3K/Akt branches (20, 29, 92). ERK1/2 and Akt mutually reinforce NF-κB activation through IKKα phosphorylation, while p38 MAPK amplifies cytokine output post-transcriptionally by stabilizing AU-rich element–containing mRNAs encoding TNF-α, IL-6, CXCL8, and COX-2 (121, 125, 126). STAT6, activated downstream of IL-4/IL-13, antagonizes STAT1-driven antiviral programs through SOCS1 induction, while synergizing with NF-κB to drive the T2-high transcriptional signature (150, 151). C/EBPβ integrates convergent outputs from ERK1/2 and STAT3 to selectively regulate chemokine genes, including CCL20, that are not fully explained by NF-κB or AP-1 alone (93, 163, 164). TGF-β/SMAD signaling further intersects the network through TAK1-mediated p38 and NF-κB co-activation, directly linking the allergen-triggered network to chronic structural outputs (159). These interconnections collectively explain emergent properties of the allergen-exposed epithelium: the co-induction of inflammatory and structural programs, the amplification loops that sustain chronic activation, and the limited efficacy of single-pathway inhibition in a subset of patients (29).

## 5 Epithelial-derived alarmins and mediator networks linking inflammation to airway remodeling

### 5.1 Epithelial alarmins as upstream initiators of type 2 airway inflammation

Airway epithelial cells are not merely passive barriers but active regulators of asthma pathobiology. In response to allergens, infections, and epithelial injury, they release alarmins, chemokines, and remodeling-associated mediators that collectively initiate inflammation, shape asthma endotypes, and promote chronic structural changes in the airway wall (167). TSLP, IL-33, and IL-25 are key epithelial alarmins that function as upstream initiators of type 2 airway inflammation (168). Released in response to allergen exposure, barrier disruption, and cellular stress, these cytokines cooperatively activate ILC2s and amplify downstream Th2 responses (168). TSLP promotes dendritic cell-driven Th2 polarization, enhances mast cell activation, and augments ILC2-derived cytokine production, underscoring its upstream role in asthma pathogenesis (169). IL-33, released from injured epithelial cells, is a potent activator of ILC2s and is strongly supported by asthma susceptibility loci identified in genetic studies. IL-25, produced mainly by tuft and goblet cells, preferentially amplifies adaptive Th2 immunity (170). Together, these alarmins establish feed-forward inflammatory circuits involving ILC2s, mast cells, and eosinophils, thereby perpetuating epithelial injury and barrier dysfunction.

### 5.2 Chemokine programs in immune cell recruitment and asthma endotype polarization

Following alarmin release, the airway epithelium directs inflammatory cell recruitment through selective chemokine production. In T2-high asthma, STAT6-driven CCL11 (eotaxin-1) and CCL24 (eotaxin-2) recruit CCR3+ eosinophils, while CCL17 and CCL22 recruit CCR4+ Th2 cells (171). In T2-low/neutrophilic asthma, IL-17A and TNF-α-driven NF-κB activation induces CXCL1, CXCL2, CXCL5, and CXCL8, recruiting CXCR1/2+ neutrophils, while IFN-γ-driven STAT1 induces CXCL9/10/11, recruiting CXCR3+ Th1 cells (172). The specific chemokine profile of the allergen-activated epithelium is thus a molecular determinant of asthma endotype. CCL20 (MIP-3α) is the sole endogenous ligand for CCR6 and links allergen-induced EGFR/MAPK/PI3K/NF-κB/C/EBPβ signaling to dendritic Cell (DC)-dependent immune amplification (173). Moreover, CCL20 performs a particularly important intermediary role by recruiting CCR6+ dendritic cells and Th17 cells, thereby linking epithelial activation to neutrophilic immune amplification (174). Thus, the epithelial chemokine milieu is a major determinant of immune-cell composition and asthma endotype polarization.

## 6 Viral exacerbations as secondary amplifiers of epithelial signaling networks

### 6.1 Respiratory viruses as exacerbation triggers and network amplifiers

Respiratory viral infections are major triggers of acute asthma exacerbations, with rhinovirus representing the most common pathogen (175). Rather than initiating a wholly distinct pathogenic program, viral infection amplifies pre-existing epithelial signaling abnormalities, thereby worsening barrier dysfunction, inflammatory mediator release, and airflow limitation (175). This explains why viral infections account for more than half of asthma exacerbations in adults and up to 80% in children (176). Respiratory viruses activate epithelial pattern-recognition pathways that converge on IRF3/7, NF-κB, and MAPK signaling, thereby inducing both antiviral interferons and pro-inflammatory mediators (177). In asthma, however, type I and type III interferon responses are often impaired or delayed, resulting in inadequate antiviral defense accompanied by exaggerated inflammation (178). Viral infection therefore acts as a potent amplifier of epithelial cytokine, chemokine, and alarmin production in an already primed airway epithelium (179).

### 6.2 The double-hit model of viral infection and allergen exposure

A useful mechanistic framework is the “double-hit” model, in which viral infection and allergen exposure converge on the same epithelial signaling circuitry to induce supra-additive inflammation (180). Viral infection increases epithelial permeability and amplifies inflammatory signaling, whereas pre-existing type 2 (T2) inflammation can further impair antiviral competence (181). This bidirectional interaction likely underlies the marked severity of exacerbations observed in sensitized patients during viral infection seasons (182). In this context, upstream biologics, such as dupilumab and tezepelumab, may help normalize the epithelial environment and partially restore antiviral defense, although this remains an area of active investigation (183).

Taken together, these findings support the view that respiratory viral infection functions less as an independent pathogenic program than as a secondary amplifier of pre-existing epithelial network dysfunction in asthma. By converging with allergen-driven signaling on shared epithelial pathways, viral infection magnifies barrier disruption, inflammatory mediator release, and airflow limitation, particularly in patients with an already primed T2 inflammatory milieu.

## 7 Therapeutic reprogramming of the asthmatic epithelium: from inflammatory blockade to steroid resensitization and barrier restoration

**Figure 4:**
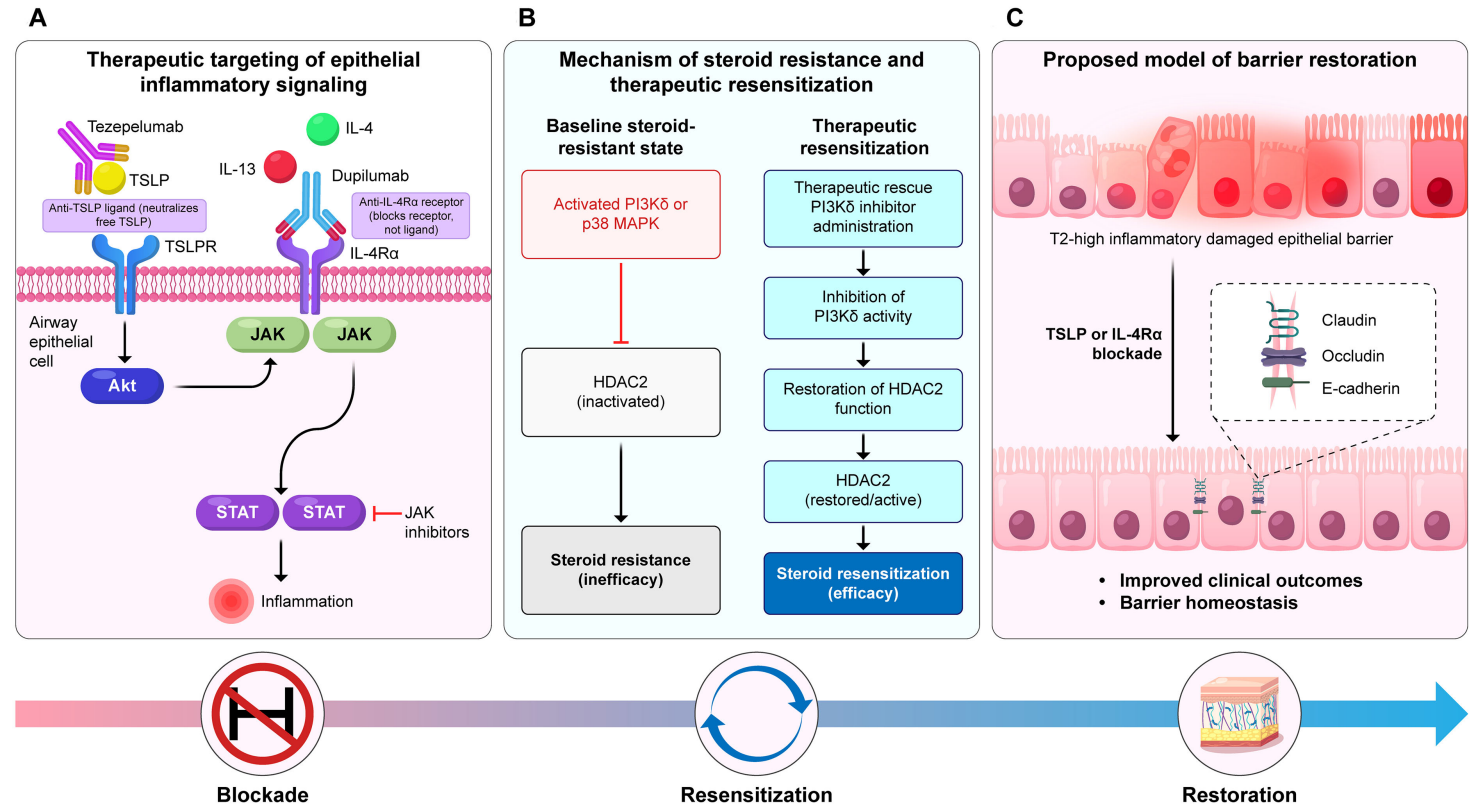
Integrated therapeutic framework of epithelial-targeted intervention in asthma: blockade, resensitization, and restoration. **(A)** Therapeutic targeting of epithelial inflammatory signaling. Tezepelumab is an anti-TSLP monoclonal antibody that neutralizes TSLP extracellularly by binding the free ligand before it engages TSLPR, thereby broadly attenuating downstream type 2 immune activation. Dupilumab, by contrast, targets the IL-4Rα receptor subunit on the cell surface, simultaneously blocking IL-4– and IL-13–mediated signaling and disrupting the JAK1/STAT6 amplification axis that sustains T2-high transcriptional programs and STAT6-dependent barrier dysfunction. JAK inhibitors provide an additional option for suppressing STAT-mediated signaling downstream of multiple cytokine receptors. Together, these agents bracket the alarmin-to-cytokine signaling axis at distinct network positions, with tezepelumab at the extracellular ligand level and dupilumab at the transmembrane receptor level, explaining differences in their breadth of T2 inflammatory inhibition. **(B)** Mechanism of corticosteroid resistance and therapeutic Resensitization. Hyperactivation of PI3Kδ or p38 MAPK, driven by sustained allergen-induced signaling and oxidative stress, contributes to HDAC2 inactivation and consequent corticosteroid inefficacy in severe asthma. Therapeutic PI3Kδ inhibition restores HDAC2 catalytic activity and promotes corticosteroid resensitization, as shown in preclinical models. Additional resensitization strategies include NRF2-activating agents that counter oxidative stress-mediated HDAC2 nitrosylation and low-dose theophylline that restores HDAC2 activity independently of PI3K. **(C)** Proposed model of barrier restoration. In T2-high inflammation, sustained IL-4/IL-13 signaling maintains epithelial barrier dysfunction through JAK1/STAT6-dependent suppression of claudin-1, claudin-18, and occludin, and upregulation of leaky claudin-2. Blockade of TSLP or IL-4Rα is proposed to facilitate recovery of barrier integrity, as indicated by improved organization of claudin, occludin, and E-cadherin. In addition, TGF-β1 from allergen-activated epithelial cells drives subepithelial fibroblast-to-myofibroblast differentiation and collagen deposition, while IL-13 and EGFR/ERK1/2 signaling promote airway smooth muscle expansion and contractility. Upstream biologic blockade is therefore expected to indirectly attenuate these structural cell responses, although direct evidence for remodeling reversal requires further prospective investigation. Together, the three panels illustrate a stepwise therapeutic progression from inflammatory blockade to corticosteroid resensitization and ultimately to barrier and structural restoration.

If viral exacerbations intensify disease primarily by amplifying pre-existing epithelial dysregulation, then effective intervention in asthma must extend beyond suppression of individual inflammatory mediators alone and instead address the broader network abnormalities that sustain epithelial activation, steroid insensitivity, and impaired tissue recovery. From this perspective, therapeutic reprogramming of the asthmatic epithelium can be viewed as a layered process encompassing upstream inflammatory blockade, restoration of corticosteroid responsiveness, and ultimately recovery of epithelial barrier integrity. This therapeutic concept is summarized in **Figure 4**, which presents a stepwise framework of epithelial-targeted intervention in asthma, progressing from upstream inflammatory blockade to corticosteroid resensitization and ultimately to barrier restoration.

### 7.1 Upstream blockade of epithelial inflammatory signaling

Recognition of the airway epithelium as an active signaling hub has important therapeutic implications in asthma, because epithelial-derived alarmins and cytokine-amplifying pathways represent accessible points of intervention (184, 185). Biologic therapies targeting upstream epithelial inflammatory axes have provided proof of concept that interruption of proximal signaling events can attenuate downstream disease activity (14, 186). **Table 1** summarizes key approved and investigational agents targeting the epithelial signaling network described in this review, organized by molecular target and current development stage. Tezepelumab, which neutralizes TSLP at the apex of the epithelial alarmin cascade, represents the most upstream biologic intervention currently approved for asthma and is unique in its ability to suppress all three major alarmin axes (TSLP, IL-33, IL-25) indirectly through reduction of T2 amplification loops (187, 188). Dupilumab, by contrast, targets IL-4Rα to simultaneously block both IL-4 and IL-13 signaling, operating downstream of alarmin release but disrupting a central cytokine amplification axis that links epithelial activation to Th2 polarization, IgE synthesis, eosinophil survival, and barrier dysfunction (189). Although their network positions differ, both agents have demonstrated direct effects on the airway epithelium: tezepelumab reduces airway submucosal eosinophilic infiltration, mucus plugging, and airway hyperresponsiveness as demonstrated in bronchial biopsy studies from the CASCADE trial (190), while dupilumab suppresses IL-4/IL-13-driven JAK1/STAT6 signaling that mediates tight junction disruption, including claudin-18 downregulation and barrier permeability, in bronchial epithelial cells (87, 191). These epithelial-level effects may contribute to clinical benefit beyond immune cell suppression alone. In addition, pharmacological inhibition of downstream JAK/STAT signaling further supports the therapeutic relevance of disrupting the epithelial-immune amplification axis, complementing the proximal blockade strategies depicted in **Figure 4A**.

**Table 1:** Approved and investigational therapeutic agents targeting components of the allergen-driven airway epithelial signaling network in asthma.^e^.

Target	Representative agent(s)	Asthma-relevant evidence	Development stage^d^	Ref
TSLP	Tezepelumab	NAVIGATOR Phase III: exacerbation reduction across all endotypes	Approved^a^	(187)
IL-4Rα	Dupilumab	LIBERTY ASTHMA QUEST Phase III: ↓exacerbations, ↑FEV_1_	Approved^a^	(188, 189)
IL-5/IL-5Rα	Mepolizumab Benralizumab	Phase III: ↓ exacerbations in T2-high eosinophilic asthma	Approved^a^	(14)
IgE	Omalizumab	Phase III: ↓ exacerbations in allergic asthma	Approved^a^	(14)
Inhaled JAK1	AZD0449 AZD4604	Phase I in T2-high asthma	Phase I	(152, 153)
PI3Kδ	Nemiralisib	Phase II asthma RCT (discontinued); preclinical class effect of steroid resensitization	Phase II (discontinued for asthma)^c^	(131, 137)
P38α MAPK	Losmapimod	Phase II in COPD (negative for primary endpoints); exploratory subgroup signals	Phase II	(127, 129, 130)
EGFR	Gefitinib^b^, Afatinib^b^	Preclinical: ↓ goblet cell metaplasia, ↓ remodeling features	Preclinical	(109, 115, 119)
TGF-β	Fresolimumab Pirfenidone	Preclinical efficacy in asthma models; approved in IPF	Preclinical (asthma)/Approved (IPF)	(37, 38, 161)
NF-κB/IKKβ	IMD-0354, TPCA-1 PHA-408	Preclinical: ↓ inflammatory gene expression	Preclinical	(138–140, 144, 145)
HDAC2	Low-dose theophylline	Mechanistic clinical studies in COPD: HDAC2 restoration	Mechanistic clinical	(199, 200)

^a^ Approved indicates regulatory approval by the U.S. Food and Drug Administration and/or the European Medicines Agency for the asthma indication.

^b^ Agents approved for non-asthma indications, including gefitinib and afatinib (non–small cell lung cancer) and pirfenidone (idiopathic pulmonary fibrosis), are included on the basis of mechanistic rationale and preclinical asthma evidence; their use in asthma remains investigational and is not endorsed for off-label clinical application.

^c^ Phase II (discontinued) indicates that the asthma-specific development program has been formally suspended or terminated by the sponsor; the underlying molecular target may nonetheless remain of continued mechanistic and therapeutic interest.

^d^ Development stages were verified against ClinicalTrials.gov, the EU Clinical Trials Register, and peer-reviewed publications available at the time of manuscript preparation; readers are encouraged to consult these registries for the most current status.

^e^ This table is not intended to provide treatment recommendations. Clinical management decisions should be guided by current asthma management guidelines (e.g., the Global Initiative for Asthma [GINA]) and by the prescribing information of regulatory-approved agents.

COPD, chronic obstructive pulmonary disease; EGFR, epidermal growth factor receptor; FeNO, fractional exhaled nitric oxide; FEV_1_, forced expiratory volume in 1 second; HDAC2, histone deacetylase 2; IKK, IκB kinase; IL, interleukin; IPF, idiopathic pulmonary fibrosis; JAK, Janus kinase; MAPK, mitogen-activated protein kinase; NF-κB, nuclear factor kappa B; PI3Kδ, phosphoinositide 3-kinase delta; RCT, randomized controlled trial; STAT, signal transducer and activator of transcription; T2, type 2; TGF-β, transforming growth factor beta; TSLP, thymic stromal lymphopoietin.

### 7.2 Corticosteroid resistance and therapeutic resensitization

Upstream inflammatory blockade alone is unlikely to fully resolve the epithelial abnormalities that sustain chronic disease, particularly in severe or treatment-refractory asthma. Within this framework, corticosteroid resistance represents a critical intermediate layer linking incomplete inflammatory control to impaired epithelial recovery. Sustained PI3Kδ/Akt signaling, oxidative stress, and p38 MAPK activation impair glucocorticoid receptor function and reduce HDAC2-dependent transcriptional repression, thereby uncoupling corticosteroid binding from effective suppression of inflammatory gene expression (192, 193). These abnormalities are particularly relevant in severe and neutrophilic asthma, in which NF-κB-, IL-17-, and oxidative stress-dominant signaling sustains epithelial activation despite corticosteroid exposure (194, 195).

Restoration of corticosteroid responsiveness may therefore require targeting the signaling hubs that maintain steroid-refractory epithelial activation rather than escalating corticosteroid dose alone. PI3Kδ inhibition is one of the most compelling resensitization strategies, as preclinical studies and early clinical investigation suggest that suppression of PI3Kδ activity may restore HDAC2 function, reduce inflammatory signaling, and improve corticosteroid sensitivity, although definitive validation in severe asthma remains incomplete (196). Although **Figure 4B** emphasizes the PI3Kδ–HDAC2 axis as the prototypic resensitization mechanism, additional resensitization strategies are also relevant, including NRF2-activating agents and low-dose theophylline that counter oxidative stress, as well as p38 MAPK inhibition that has been implicated in glucocorticoid receptor dysfunction (127, 137, 197–200). Because these pathways may be differentially active across asthma endotypes, rational selection of single agents or combination strategies may offer a more effective route to steroid resensitization than dose escalation alone.

### 7.3 Barrier restoration as the therapeutic endpoint

Corticosteroid resistance is not merely a therapeutic obstacle but a mechanistic bridge between persistent epithelial inflammation and failure of tissue recovery (201, 202). Accordingly, strategies that restore corticosteroid responsiveness may facilitate not only improved suppression of inflammatory gene expression but also recovery of epithelial barrier integrity (201). From a network-based perspective, the therapeutic goal should extend beyond inflammation control to restoration of epithelial homeostasis (36). Chronic epithelial activation contributes to junctional disruption, mucous differentiation, and remodeling-associated dysfunction, and successful treatment should therefore be judged in part by recovery of epithelial integrity and tissue behavior (203).

The therapeutic framework discussed in this section has important implications that extend beyond epithelial and immune cell biology to encompass structural airway remodeling driven by fibroblasts and airway smooth muscle (ASM) cells (4). TGF-β1, released by allergen-activated epithelial cells and further amplified by the signaling network described throughout this review, is the central paracrine signal driving subepithelial fibroblast-to-myofibroblast differentiation, collagen I/III deposition, and reticular basement membrane thickening, all of which are hallmarks of irreversible structural remodeling in severe asthma (118, 135). IL-13, acting through JAK1/STAT6 in ASM cells, promotes both contractility and proliferative expansion (160), while EGFR-linked ERK1/2 signaling in ASM drives cell-cycle progression independently of cytokine input (204). Upstream biologic blockade of TSLP or IL-4Rα is therefore expected to indirectly attenuate these structural cell responses by reducing the epithelial-derived cytokine milieu available to fibroblasts and ASM, although direct, biopsy-confirmed evidence for structural remodeling reversal with current biologics remains an active area of clinical investigation and a critical unmet endpoint (183).

Barrier restoration is thus not simply a downstream consequence of reduced inflammation, but a central endpoint of epithelial disease modification (205). Re-establishment of junctional stability, including improved organization of claudin, occludin, and E-cadherin-associated structures, may indicate that pathogenic epithelial signaling has been durably attenuated and that the tissue is transitioning toward functional recovery (**Figure 4C**) (191). Taken together, these observations support a layered therapeutic model in which effective epithelial reprogramming proceeds from inflammatory blockade to steroid resensitization and ultimately to barrier restoration, as collectively summarized in **Figure 4**.

Importantly, this layered therapeutic logic is meaningful only when interpreted within the broader signaling architecture introduced earlier in this review. The same network features that explain why single-target interventions are often insufficient also define the rational basis for sequencing blockade, resensitization, and barrier restoration as complementary rather than competing strategies. From this vantage point, the therapeutic implications discussed above naturally extend into a wider conceptual synthesis of how the asthmatic epithelium should be understood as a disease-defining entity.

## 8 Discussion and future directions

This review positions the allergen-exposed airway epithelium as the central molecular hub of asthma pathogenesis in allergic asthma, and as the common origin of both acute inflammatory and chronic structural outputs arising from a single integrated signaling network. Through convergent activation of EGFR, MAPK, PI3K/Akt, NF-κB, JAK/STAT, and TGF-β/SMAD pathways, each with defined mechanistic links to specific remodeling phenotypes, the allergen-exposed epithelium simultaneously drives innate immune activation and initiates the structural changes that define progressive disease. Specifically: sustained EGFR/ERK1/2 signaling drives SPDEF-dependent goblet cell transdifferentiation and mucous metaplasia; PI3K/Akt–mediated Snail stabilization and GSK-3β inhibition initiate EMT with loss of epithelial junctional integrity; TGF-β/SMAD-driven SMAD2/3 transcription activates subepithelial fibroblast-to-myofibroblast differentiation, collagen I/III deposition, and reticular basement membrane thickening; and IL-13/STAT6 signaling, amplified by JAK1, promotes airway smooth muscle proliferation and contractile hypertrophy (29, 206). These pathway-to-phenotype linkages are not parallel but convergent: the same allergen exposure event simultaneously activates all of these axes through the interconnected epithelial signaling network described throughout this review, explaining why inflammation and remodeling co-occur, mutually amplify, and progress together in chronic asthma. This network perspective explains several clinically important observations: the co-occurrence and mutual amplification of inflammation and remodeling, the limited disease-modifying capacity of single-target interventions in a subset of patients, and the progressive, epigenetically consolidated nature of severe asthma.

A central conceptual contribution of this review is the unified network model of allergen-induced epithelial signaling. The extensive crosstalk among core signaling pathways, further shaped by C/EBPβ-mediated transcriptional specificity, epigenetic consolidation, viral exacerbation-mediated amplification, and molecular mechanisms of corticosteroid resistance, generates an integrated signaling landscape from which both inflammatory and structural outputs are simultaneously produced. Within this framework, distinct asthma endotypes can be reinterpreted as alternative configurations of a shared epithelial network rather than as biologically separate diseases (206), with their phenotypic divergence shaped by upstream allergen, microbial, and viral inputs.

These mechanistic insights have direct therapeutic implications. In the short term, upstream cytokine-directed biologics and emerging anti-ST2/anti-IL-33 agents represent validated and expanding options (187), whereas PI3Kδ inhibition and HDAC2 reactivation constitute rational approaches for steroid-resistant disease (207–209). In the medium to long term, inhaled JAK1 inhibitors, barrier-restoration strategies, and rational combination regimens targeting multiple network hubs offer the most promising directions for achieving comprehensive disease modification.

Several important questions remain to be addressed. The precise hierarchy of signaling crosstalk within the epithelial network, the mechanisms by which epigenetic memory is established and potentially reversed, and the cell-type-specific contributions of distinct epithelial populations including tuft cells and ionocytes all warrant systematic investigation. The development of validated biomarkers reflecting active remodeling and enabling patient stratification for network-targeted interventions also represents a critical unmet need. Ultimately, the convergence of multi-omics profiling, CRISPR-based functional screening, airway organoid systems, and biomarker-enriched clinical trial design offers a realistic path toward truly disease-modifying therapy capable of reversing or preventing established structural airway changes in asthma.
